# No evidence that serum metabolite slopes improve held-out prediction of clinical progression in the Alzheimer’s disease neuroimaging initiative

**DOI:** 10.3389/fnagi.2026.1942662

**Published:** 2026-10-01

**Authors:** Tia Warrick, Manali Misra, Stephen Kataria, Jahnavi Patel, Bhuvi Ajmera

**Affiliations:** Department of Health Sciences, Juniata College, Huntingdon, PA, United States

**Keywords:** ADNI, Alzheimer’s disease, APOE, clinical progression, longitudinal biomarkers, metabolomics, prediction model, validation

## Abstract

**Background:**

Repeated serum metabolite measurements may contribute to predicting clinical progression, but their incremental value requires evaluation beyond established clinical predictors.

**Methods:**

We linked Alzheimer’s Disease Neuroimaging Initiative (ADNI) clinical data to Biocrates Quant 500 serum measurements. Annualized metabolite slopes were estimated from baseline through month 12, usually from two observations. At the month-12 landmark, participants without dementia were characterized by diagnosis, memory performance, and APOE ε4 status; progression was assessed at approximately month 36. Models were developed in ADNI1 (225 participants; 26 events) and applied without refitting to ADNI-GO/2 (269 participants; 50 events). We compared a clinical logistic model with a model that added a data-derived phosphatidylcholine (PC) aa C42:1 candidate slope. Models using 88 phosphatidylcholine-family metabolite slopes or 598 full-panel slopes were exploratory.

**Results:**

The clinical model achieved an area under the receiver operating characteristic curve (AUROC) of 0.758 (95% CI, 0.675–0.838). Adding PC aa C42:1 changed the AUROC by +0.0004 (−0.013 to 0.017). Both partially penalized lasso models retained no metabolite slopes at the selected penalty. A *post-hoc* evaluation restricted to 233 participants with mild cognitive impairment, including 38 events, also found no evidence of incremental discrimination. Other sensitivity analyses yielded similar results.

**Conclusion:**

We found no evidence that annualized serum metabolite slopes improved held-out prediction in this sample. The 26 development events limited estimation and feature selection, so these results cannot distinguish between a lack of incremental prognostic information and insufficient data to identify such information. Larger development samples and independent validation are needed to assess the value of serial metabolomics.

## 1 Introduction

Alzheimer’s disease and related cognitive syndromes progress at markedly different rates across individuals. This heterogeneity complicates counseling, monitoring, and clinical-trial enrollment and has motivated prognostic models that combine clinical assessments with biomarkers (Aisen et al., 2024; Jack et al., 2018; Petersen et al., 2010). Blood-based biomarkers increasingly inform Alzheimer’s research and care (Hampel et al., 2023; Palmqvist et al., 2024). Their clinical value depends on the intended use: diagnostic discrimination, biological association, and prediction of future progression address different questions.

Other biomarker modalities include magnetic resonance spectroscopy (MRS) and electroencephalography (EEG). Wei et al. (2024) systematically evaluated MRS biomarkers for Alzheimer’s disease diagnosis, while Zúñiga et al. (2024) reviewed EEG entropy measures. These approaches illustrate the range of quantitative biomarkers under study, but diagnostic performance does not establish incremental prognostic value beyond clinical information. Repeated serum measurements require evaluation for that specific purpose.

Metabolomics is attractive for progression research because circulating metabolites reflect dynamic biochemical states influenced by physiology, diet, medications, and disease. Lipid metabolism, including phosphatidylcholines (PCs), has been associated with amyloid deposition, cognitive performance, neurodegeneration, and dementia progression in Alzheimer’s Disease Neuroimaging Initiative (ADNI) and related cohorts (Barupal et al., 2019; Horgusluoglu et al., 2022; Lista et al., 2023; Nho et al., 2021; Shanks et al., 2022; Yin et al., 2023). Most reported associations, however, are based on single measurements or evaluate performance in the same cohort used to select biomarkers. Serial measurements add assay burden and cost, so their value should be judged by incremental performance in participants not used for feature selection or model tuning.

PC aa C42:1 was selected as a data-derived candidate because it emerged as the leading analyte in an earlier false-discovery-rate-controlled screen of unsaturated PC-family species. That screen used overlapping ADNI data, including ADNI1, and was not an independent discovery cohort. The candidate was prespecified before the ADNI-GO/2 performance evaluation. Its prioritization was also consistent with prior ADNI studies linking phosphatidylcholine dysregulation with amyloid deposition, cognition, neurodegeneration, and dementia progression (Barupal et al., 2019; Nho et al., 2021; Shanks et al., 2022). We evaluated this hypothesis-generating candidate using a fixed outcome horizon and separate model-development and evaluation phases.

We examined whether annualized serum metabolite slopes added predictive information beyond landmark diagnosis, memory performance, and APOE ε4. The primary comparison evaluated the addition of PC aa C42:1 to a compact clinical model. Exploratory analyses considered 88 eligible PC-family slopes and 598 slopes across the full Quant 500 flow-injection analysis (FIA) and ultra-performance liquid chromatography (UPLC) panels using partially penalized lasso regression and a random forest. With only 26 development events, these analyses could assess the performance of the fitted models but could not reliably exclude incremental value that a larger sample might identify. Reporting followed established principles for prediction-model evaluation, including held-out discrimination, calibration, and uncertainty (Collins et al., 2024; Riley et al., 2019; Van Calster et al., 2019; Wolff et al., 2019).

## 2 Materials and methods

### 2.1 Study design and data sources

Data used in the preparation of this article were obtained from the Alzheimer’s Disease Neuroimaging Initiative (ADNI) database (adni.loni.usc.edu). The ADNI was launched in 2003 as a public–private partnership, led by Principal Investigator Michael W. Weiner, MD. The primary goal of ADNI has been to determine whether serial magnetic resonance imaging, positron emission tomography, other biological markers, and clinical and neuropsychological assessments can be combined to measure the progression of mild cognitive impairment and early Alzheimer’s disease.

This secondary analysis linked ADNI diagnosis records, the ADNI-MEM composite, APOE genotypes, and serum metabolomics files generated by the Alzheimer’s Disease Metabolomics Consortium (ADMC). The metabolomics platform was the Biocrates MxP Quant 500 assay, with data represented in longitudinal FIA and UPLC tables. We used a fixed-landmark design with temporally ordered predictors and outcomes: metabolite measurements from baseline through month 12 formed the predictor window, clinical predictors were ascertained near month 12, and subsequent diagnostic status was assessed near month 36. The 12-month predictor window captured approximately 1 year of repeated metabolite information while keeping predictor ascertainment separate from the subsequent risk period.

Before cohort construction, records from PTIDs beginning with 381_S_ were excluded in accordance with the March 2026 ADNI Data Advisory (Alzheimer’s Disease Neuroimaging Initiative, 2026). The diagnosis source contained 139 records from 78 affected participants. Neither Quant 500 source file contained records with this affected prefix; therefore, no affected metabolomics record entered the analysis.

### 2.2 Landmark, outcome, and cohort separation

The earliest valid diagnosis record defined ADNI baseline. For each participant, the diagnosis nearest month 12 was selected within an eligible window of months 8–16. Participants with dementia at the landmark were excluded. The outcome was the diagnosis nearest month 36 within months 30–42 and was required to occur after the landmark.

Diagnosis was harmonized as cognitively normal, mild cognitive impairment (MCI), or dementia. Progression was defined as a transition from cognitively normal to MCI or dementia, or from MCI to dementia. The endpoint therefore represents syndromal diagnostic progression and not biomarker-confirmed Alzheimer’s disease progression. Because transitions from CN to MCI and from MCI to dementia transitions can occur for reasons other than Alzheimer pathology, this endpoint should not be interpreted as etiologically specific to Alzheimer’s disease.

ADNI1 was used exclusively for model fitting, preprocessing estimation, feature eligibility, and hyperparameter tuning. ADNI-GO and ADNI2 were combined as a locked held-out evaluation cohort. All models were applied to ADNI-GO/2 without refitting or recalibration. This phase-separated design provides a held-out internal-temporal evaluation within ADNI rather than an external validation in an independent cohort.

### 2.3 Clinical predictors

The clinical model contained landmark diagnosis (MCI vs. cognitively normal), the ADNI-MEM score nearest month 12 within the same 8–16-month window, and APOE ε4 carrier status. ADNI-MEM is a validated composite that harmonizes multiple memory measures across ADNI phases (Crane et al., 2012). APOE ε4 carrier status was derived from the first available genotype record. The primary benchmark was limited to these three consistently harmonized predictors. Age was examined in a sensitivity analysis. A separate sensitivity model incorporated baseline ADNI-MEM and its annualized change over months 0–12 to evaluate whether longitudinal clinical information altered the comparison with metabolite slopes.

### 2.4 Metabolomics processing and slope features

The FIA file contained 518 candidate measurements, and the UPLC file contained 106, yielding 624 non-overlapping Quant 500 features. Duplicate participant-visit records were averaged. Visit codes were converted to months from baseline, and only measurements from months 0–12 were used.

For each metabolite, we estimated an annualized ordinary-least-squares slope against visit time in years. An estimable slope required at least two non-missing values at distinct visits spanning at least 6 months. With two observations, this slope was calculated as the concentration difference divided by elapsed time in years. Feature eligibility was determined in ADNI1 only: a slope had to be available for at least 50% of derivation participants and have non-zero variance. This rule retained 598 slopes, including 88 diacyl PC, acyl-alkyl PC, and lysophosphatidylcholine slopes. Each feature represented a linear rate of change; it did not preserve non-linear or visit-specific temporal patterns.

PC aa C42:1 was retained as the data-derived candidate analyte. In the platform nomenclature, PC aa denotes a diacyl phosphatidylcholine, and C42:1 denotes a sum composition of 42 total carbon atoms and one total double bond; exact fatty-acid and positional isomers are not resolved. Its selection preceded held-out performance evaluation but arose from the overlapping ADNI screen described above.

### 2.5 Prediction models

Five models were evaluated on a common cohort. The primary comparison was between an approximately unpenalized logistic regression using the three clinical predictors and the same model with PC aa C42:1 added. Both logistic models used a very weak L2 penalty (C = 10^6^) with the liblinear solver. Exploratory partially penalized lasso models included all clinical predictors without penalization and applied an L1 penalty to either 88 PC-family slopes or all 598 eligible Quant 500 slopes (Tibshirani, 1996). A full-panel random forest was included as an exploratory non-linear model (Breiman, 2001). The high-dimensional models were poorly supported by the 26 development events and were not treated as tests capable of excluding non-linear or multimetabolite effects.

Within each model fit, missing continuous slopes were median-imputed from the ADNI1 training data, and continuous predictors were standardized using ADNI1 estimates. Median imputation was used as a simple training-derived procedure that could be applied unchanged to the held-out cohort while avoiding evaluation-set leakage. For the partially penalized lasso models, clinical predictors were forced into the model and L1 penalization was applied only to metabolite slopes. Penalty strength was selected by five-fold stratified ADNI1 cross-validation over lambda fractions of 1, 0.5, 0.25, 0.125, 0.0625, 0.03125, and 0.015625 relative to the training-fold lambda-max. Ties in mean cross-validation area under the receiver operating characteristic curve (AUROC) were resolved in favor of the stronger penalty. The random forest used 300 trees, balanced class weights, square-root feature sampling, and three-fold stratified cross-validation over maximum depth (3 or unrestricted) and minimum leaf size (5 or 15). No outcome resampling or class weighting was applied to the logistic or lasso models; balanced class weights were retained for the random forest. No ADNI-GO/2 information was used for preprocessing or tuning.

### 2.6 Performance evaluation

The primary measure was the AUROC. We also reported the Brier score for overall probability error and average precision for precision-recall performance (Saito and Rehmsmeier, 2015). Calibration intercept and slope were estimated in the held-out cohort; ideal values are 0 and 1, respectively (Van Calster et al., 2019). Secondary metrics were interpreted descriptively alongside calibration and outcome prevalence, without claims about clinical benefit.

We estimated uncertainty with 5,000 paired, class-stratified bootstrap samples of the held-out cohort. Each resample comprised 50 progression events and 219 non-events drawn with replacement within each outcome class. We used the same resample indices for every model and for AUROC, Brier score, and average precision. Average precision was recalculated from the observed outcomes and fixed predicted probabilities in each resample; the 2.5th and 97.5th percentiles defined its 95% confidence interval. Models were not refitted during resampling. These intervals therefore describe evaluation-sample uncertainty conditional on the fitted models and observed class counts. Paired percentile intervals were calculated for AUROC and Brier-score differences relative to the clinical model. Two-sided bootstrap *p*-values were reported for AUROC differences. We did not optimize threshold-specific sensitivity or specificity because the objective was incremental prediction rather than selection of a clinical decision threshold.

### 2.7 Sensitivity analyses and software

A transformation sensitivity analysis estimated slopes after a log2 transformation of positive concentrations and repeated the candidate and full-panel lasso evaluations. A quality-control sensitivity analysis scaled PC aa C42:1 values by the cohort-specific overall study-pool median divided by the plate-specific study-pool median; values below the plate-specific lower limit of detection were treated as missing before reconstructing the annualized slope. Additional sensitivity analyses evaluated the feasibility of a 0-6-month metabolite window, a stricter 75% feature-availability threshold, inclusion of age, a longitudinal clinical model containing baseline ADNI-MEM and its 0-12-month slope, and an outcome that counted diagnostic worsening occurring at any time after the landmark through month 42 within the common analytic cohort. We also compared the seven-value lasso grid with a four-value grid (1, 0.5, 0.25, and 0.10), using identical ADNI1 folds and preprocessing. This comparison is reported in Supplementary Table S15.

In a *post-hoc* sensitivity analysis, we restricted ADNI-GO/2 evaluation to participants with MCI at the month-12 landmark. We applied the original ADNI1-fitted models without refitting, retuning, or recalibration. The endpoint in this subgroup was MCI-to-dementia progression. We calculated performance and paired differences using 5,000 class-stratified bootstrap samples within the subgroup. This analysis assesses performance within one diagnostic stage; it does not validate a model developed specifically for MCI.

The primary analysis and preceding reproducibility checks used Python 3.12.13. The MCI-only analysis used Python 3.12.14 with the same statistical package versions: pandas 2.2.3, NumPy 2.3.5, SciPy 1.17.0, scikit-learn 1.8.0, and statsmodels 0.14.6. The random seed was 20,260,719. Supplementary Code S1 contains the primary analysis and bootstrap calculations, Supplementary Code S2 contains the additional sensitivity analyses, Supplementary Code S3 contains the penalty-grid comparison, and Supplementary Code S4 contains the held-out MCI-only evaluation.

### 2.8 Ethics statement

ADNI participants provided written informed consent under protocols approved by the institutional review board at each participating site. The present study used deidentified secondary data and required no additional participant contact. Data were analyzed under the ADNI Data Use Agreement.

## 3 Results

### 3.1 Analytic cohort

The fixed-landmark outcome was available for 764 participants, and 755 also had landmark ADNI-MEM and APOE information. Requiring an estimable PC aa C42:1 slope produced a common analytic cohort of 494 participants. ADNI1 contributed 225 participants and 26 progression events; ADNI-GO/2 contributed 269 participants and 50 events (Figure 1; Table 1).

**Figure 1:**
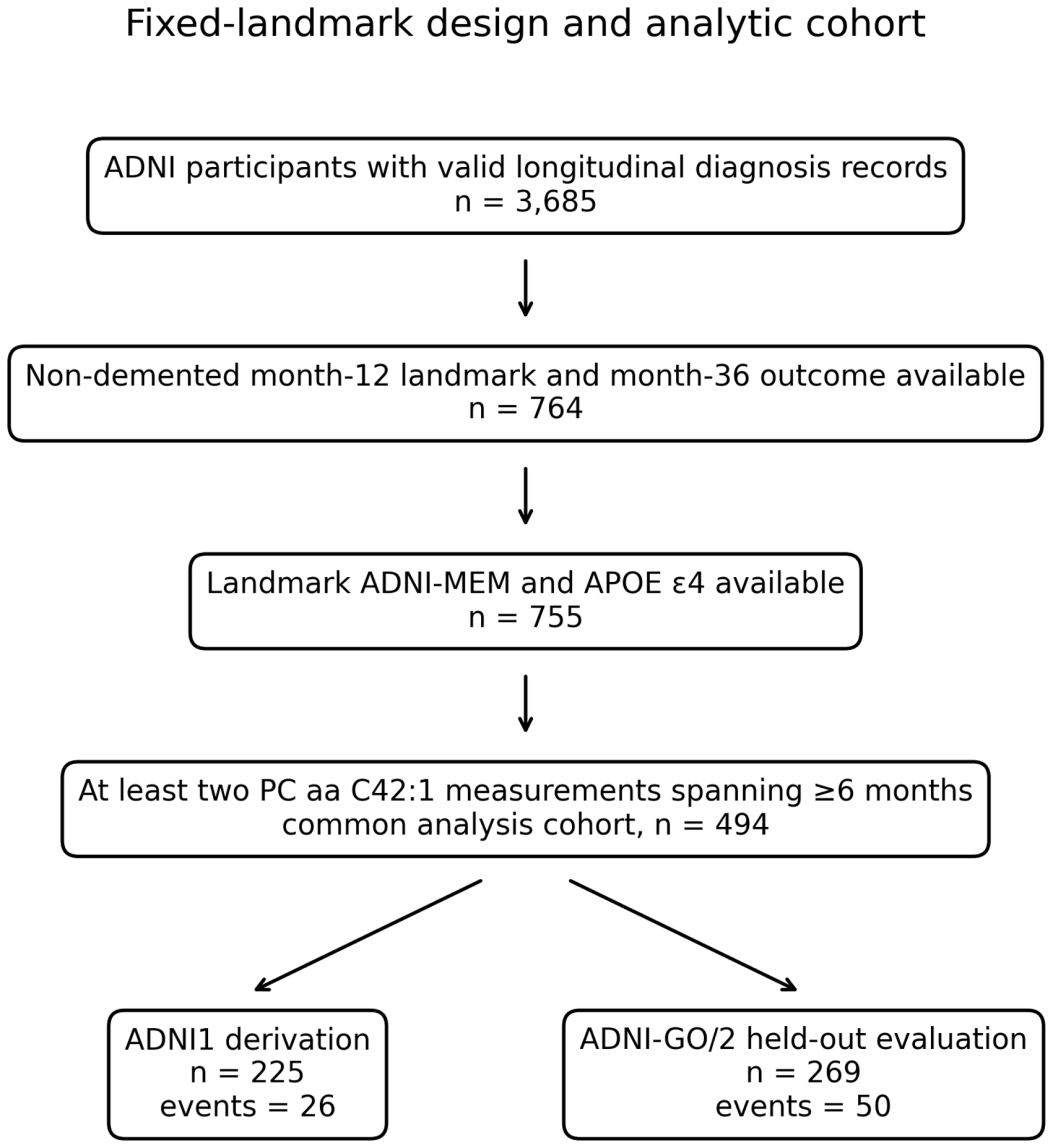
Fixed-landmark design and analytic cohort. Metabolite measurements from baseline through month 12 were used to estimate annualized slopes. Diagnosis and ADNI-MEM were fixed near month 12, and diagnostic progression was assessed near month 36. Models were developed in ADNI1 and evaluated without refitting in ADNI-GO/2.

**Table 1:** Characteristics of the derivation and held-out evaluation cohorts.

Characteristics	ADNI1 derivation	ADNI-GO/2 evaluation
Participants, *n*	225	269
Progression events, *n* (%)	26 (11.6%)	50 (18.6%)
Landmark cognitively normal, *n* (%)	117 (52.0%)	36 (13.4%)
Landmark mild cognitive impairment, *n* (%)	108 (48.0%)	233 (86.6%)
Landmark ADNI-MEM, mean (SD)	0.71 (0.90)	0.65 (0.94)
APOE ε4 carriers, *n* (%)	80 (35.6%)	107 (39.8%)
Landmark assessment, median (IQR), months	12.1 (11.8–12.5)	12.9 (12.2–13.6)
Outcome assessment, median (IQR), months	36.3 (35.9–36.7)	37.2 (36.5–38.4)
Metabolite visits in predictor window, median (IQR)	2 (2–2)	2 (2–2)
Metabolite observation span, median (IQR), months	12.0 (12.0–12.0)	12.0 (12.0–12.0)

Values are based on the common cohort with an estimable PC aa C42:1 slope. ADNI-MEM is a standardized memory composite. Diagnostic transitions are detailed in Supplementary Table S18. IQR, interquartile range.

The evaluation cohort contained a greater proportion of participants with MCI at the landmark than the derivation cohort (86.6% vs. 48.0%) and a higher overall progression frequency (18.6% vs.11.6%). Development events comprised four of 117 cognitively normal participants and 22 of 108 participants with MCI. Evaluation events comprised 12 of 36 cognitively normal participants and 38 of 233 participants with MCI (Supplementary Table S18). These events represent different diagnostic transitions. Participants in both cohorts typically contributed two metabolite measurements spanning the 12-month period.

### 3.2 Clinical model and PC aa C42:1 candidate

The locked clinical model achieved a held-out AUROC of 0.758 (95% CI, 0.675–0.838), a Brier score of 0.124, and average precision of 0.530 (95% CI, 0.410–0.671) (Table 2; Figure 2; Supplementary Figure S3). Its calibration intercept was 0.75, and its calibration slope was 0.66, indicating that absolute risk would require recalibration before the model could be used in a new setting (Figure 3).

**Table 2:** Held-out performance in ADNI-GO/2.

Model	AUROC (95% CI)	Brier score (95% CI)	Average precision (95% CI)
Clinical	0.758 (0.675 to 0.838)	0.124 (0.107 to 0.140)	0.530 (0.410 to 0.671)
Clinical + PC aa C42:1	0.759 (0.676 to 0.836)	0.126 (0.109 to 0.143)	0.515 (0.397 to 0.657)
Clinical + PC-family lasso (exploratory)	0.758 (0.675 to 0.838)	0.124 (0.107 to 0.140)	0.530 (0.410 to 0.671)
Clinical + full Quant 500 lasso (exploratory)	0.758 (0.675 to 0.838)	0.124 (0.107 to 0.140)	0.530 (0.410 to 0.671)
Clinical + full Quant 500 random forest (exploratory)	0.688 (0.604 to 0.767)	0.143 (0.136 to 0.150)	0.323 (0.253 to 0.435)

The clinical-vs.-PC aa C42:1 comparison is primary; both lasso models and the random forest are exploratory. All models were developed in ADNI1 and applied without refitting to ADNI-GO/2. Intervals are from 5,000 class-stratified bootstrap samples with fitted models fixed. The lasso models retained clinical predictors without penalization and applied L1 penalization only to metabolite slopes. Brier score and average precision are descriptive secondary metrics interpreted alongside calibration and case mix.

**Figure 2:**
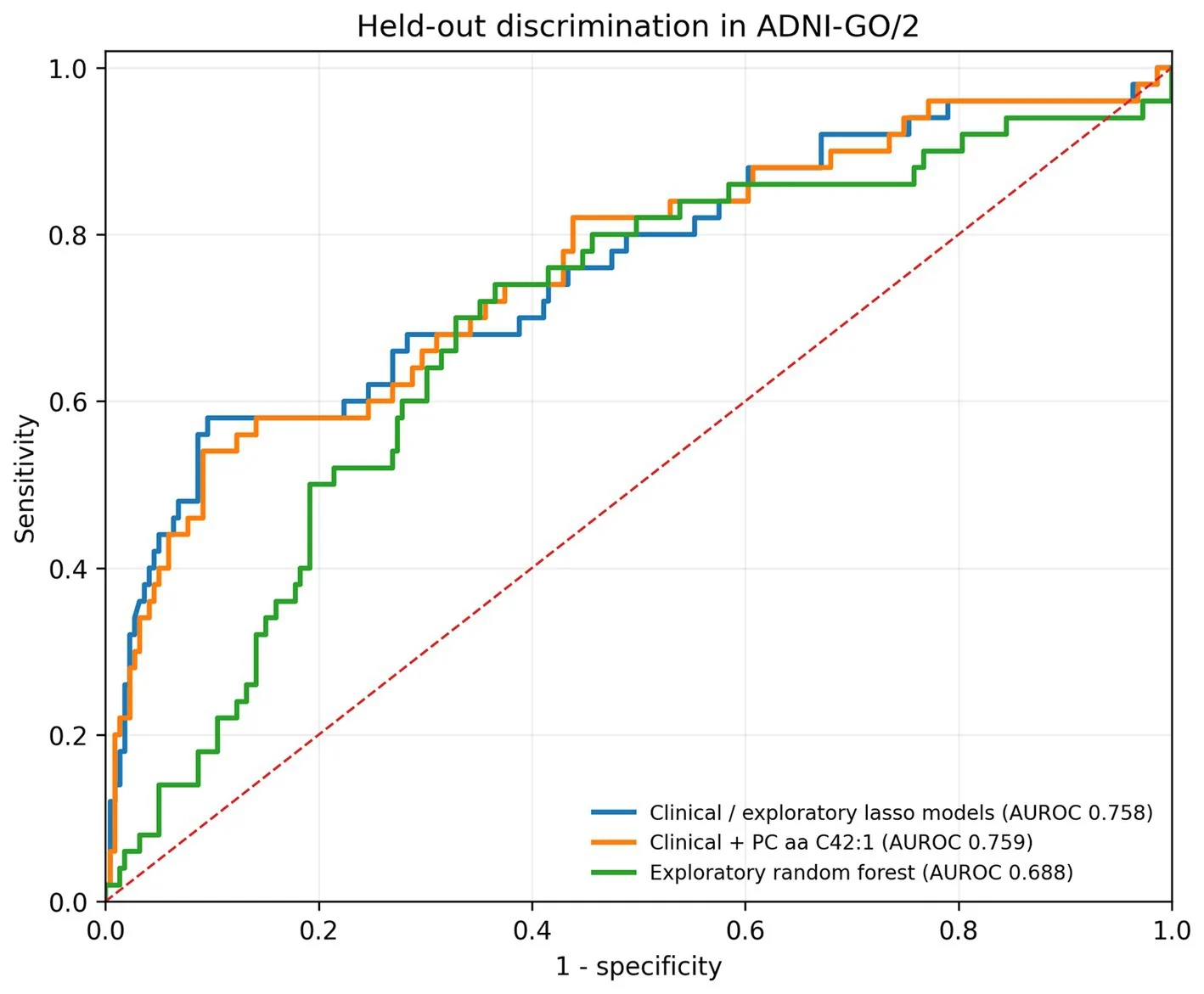
Held-out receiver operating characteristic curves. The clinical model and both exploratory lasso models produced identical held-out predictions and share one curve. Separate curves show the PC aa C42:1 candidate model and exploratory random forest in ADNI-GO/2. The random-forest comparison is not evidence against non-linear metabolite effects.

**Figure 3:**
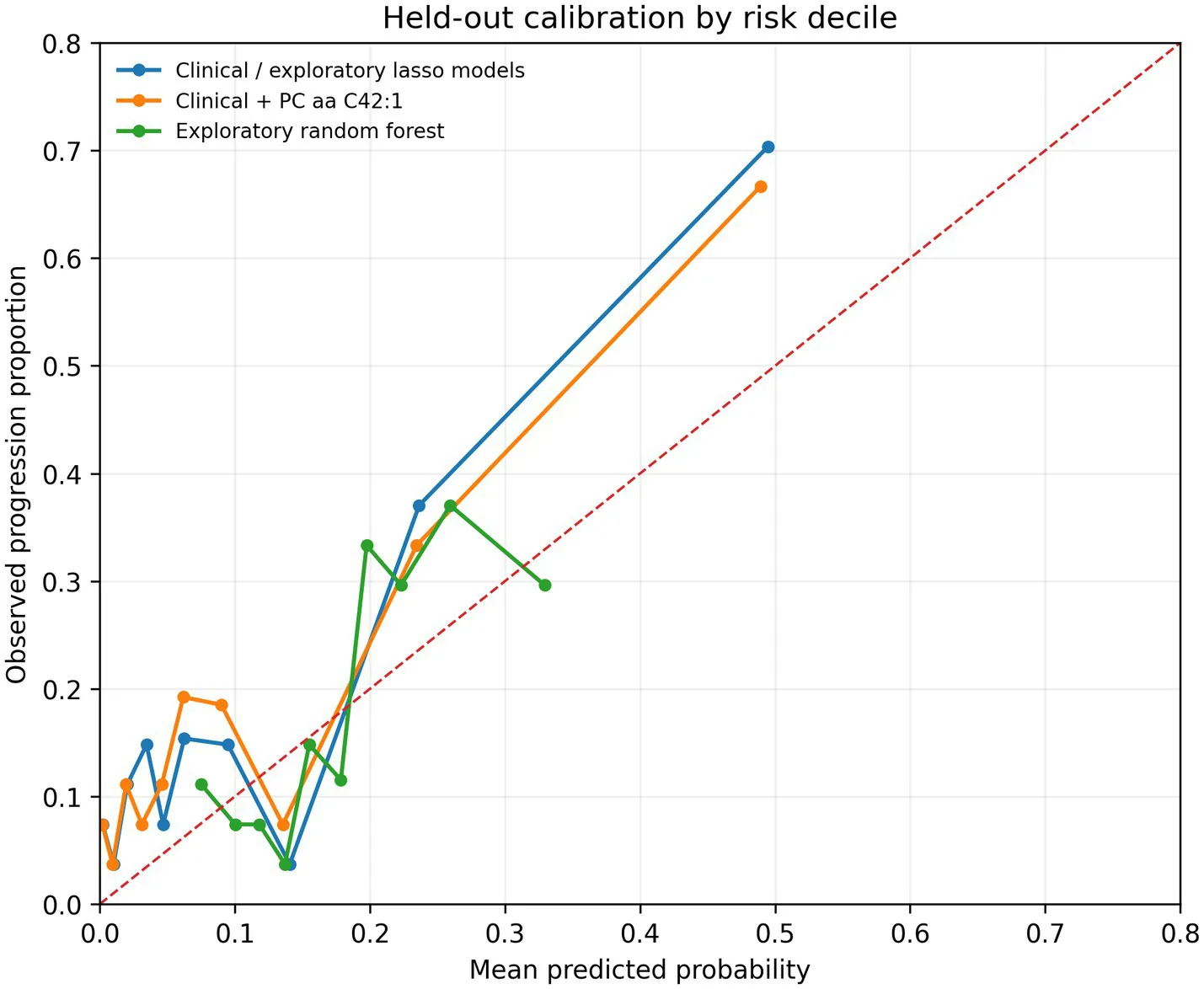
Held-out calibration by risk decile. Mean predicted probability is plotted against observed progression proportion within model-specific risk deciles. Both exploratory lasso models share the clinical-model curve because their predictions were identical. The diagonal denotes perfect calibration. Curves are descriptive because the evaluation cohort contained 50 events.

Adding PC aa C42:1 yielded an AUROC of 0.759 (95% CI, 0.676–0.836). The paired change was +0.0004 (−0.013 to 0.017; bootstrap *p* = 0.999) (Table 3; Figure 4). The Brier score was 0.0024 higher, and average precision was 0.016 lower than those of the clinical model. These secondary comparisons describe the performance of the unrecalibrated models in this cohort. In ADNI1, the standardized PC slope had an odds ratio of 0.78 (95% CI, 0.49–1.26; *p* = 0.318) in the candidate model (Supplementary Table S1).

**Table 3:** Paired changes in held-out performance relative to the clinical model.

Model	ΔAUROC (95% CI)	*p*-value	ΔBrier (95% CI)	Δ average precision
Clinical + PC aa C42:1	0.0004 (−0.0134 to 0.0168)	0.999	0.0024 (−0.0003 to 0.0051)	−0.016
Clinical + PC-family lasso (exploratory)	0.0000 (0.0000 to 0.0000)	1.000	0.0000 (0.0000 to 0.0000)	0.000
Clinical + full Quant 500 lasso (exploratory)	0.0000 (0.0000 to 0.0000)	1.000	0.0000 (0.0000 to 0.0000)	0.000
Clinical + full Quant 500 random forest (exploratory)	−0.0704 (−0.1549 to 0.0146)	0.114	0.0195 (0.0046 to 0.0346)	−0.208

Positive ΔAUROC and Δ average precision favor the metabolomic model; negative ΔBrier favors the metabolomic model. Intervals are paired percentile bootstrap intervals conditional on the fitted models. The zero-width lasso intervals reflect identical predictions, not proof that the panels contain no prognostic information. Both lasso models and the random forest are exploratory.

**Figure 4:**
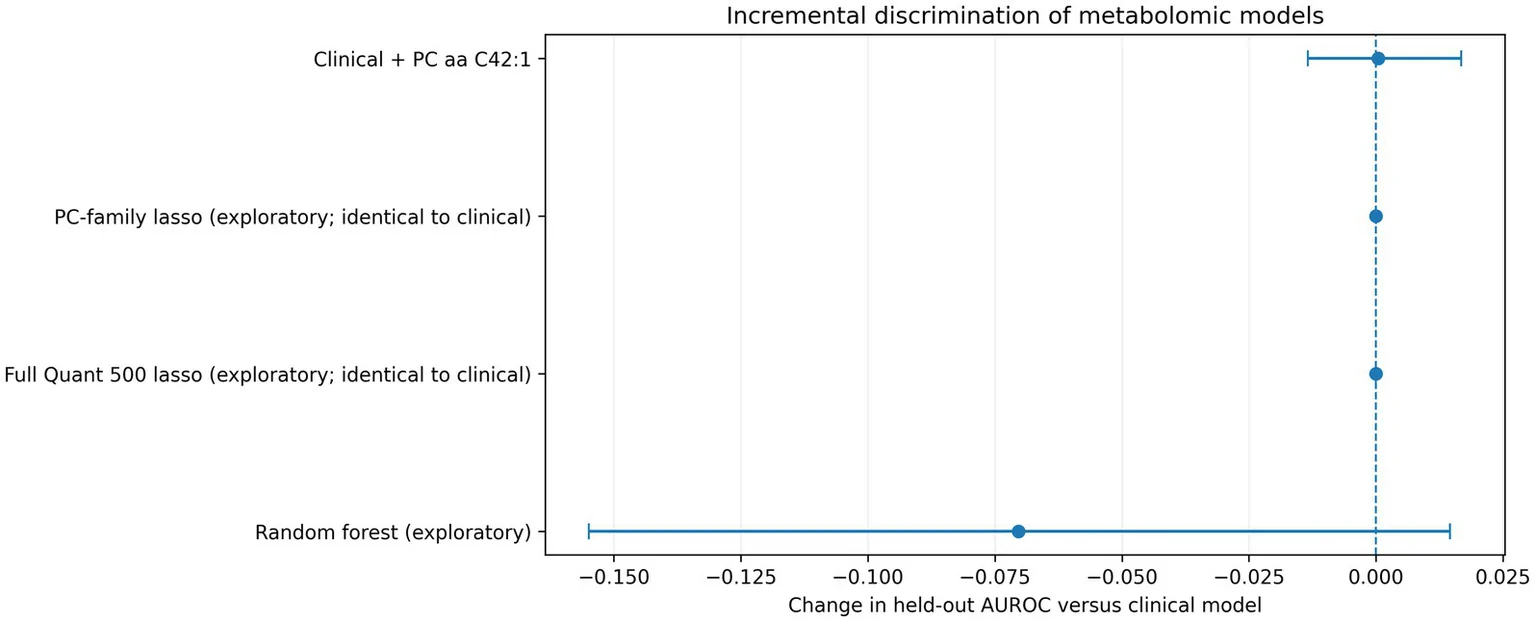
Paired changes in held-out AUROC relative to the clinical model. Points are observed differences and error bars are 95% percentile intervals from 5,000 paired class-stratified bootstrap samples. The exploratory lasso models lie at zero because their predictions reproduce the clinical model. These intervals condition on the fitted models and do not include development uncertainty. The vertical line denotes no incremental discrimination.

### 3.3 Exploratory high-dimensional models

The PC-family and full Quant 500 lasso models each selected no metabolite slopes at the cross-validated penalty while retaining the three clinical predictors. Both therefore reproduced the clinical-model predictions in held-out evaluation (Supplementary Tables S2–S4).

Selecting no slopes at this penalty describes only the behavior of the fitted models. Given the limited development sample, it does not establish that the candidate panels contain no incremental prognostic information.

The exploratory random forest achieved an AUROC of 0.688 and a Brier score of 0.143. Its average precision was 0.323 (95% CI, 0.253–0.435). Its AUROC was 0.070 lower than that of the clinical model, although the interval for the difference included zero (−0.155 to 0.015). With 598 candidate slopes and 26 development events, this comparison is vulnerable to unstable fitting and overfitting. It cannot resolve whether non-linear metabolite effects would be useful with an adequately sized development sample.

### 3.4 Sensitivity analyses

The transformation, assay-quality-control, feature-availability, age, longitudinal-memory, and early-progression analyses gave similar clinical-vs.-metabolite comparisons (Supplementary Tables S7, S8, S11–S14). The 0–6-month analysis was not fitted because only five ADNI1 participants, including one progression event, had an estimable PC aa C42:1 slope in that window. Raising the feature-availability threshold from 50 to 75% retained 591 rather than 598 slopes. Both lasso penalty grids selected 1.0 × lambda-max for both panels and yielded identical held-out predictions (Supplementary Table S15). These checks do not address uncertainty arising from the small development sample.

The MCI-only evaluation included 233 participants and 38 progression events. The clinical model achieved an AUROC of 0.860 (95% CI, 0.791–0.921), compared with 0.861 (95% CI, 0.796–0.917) after adding PC aa C42:1. The paired change was +0.0009 (−0.0159 to 0.0215). Both lasso models again reproduced the clinical-model predictions. Full subgroup results, including the exploratory random forest and secondary metrics, are provided in Supplementary Tables S16, S17.

## 4 Discussion

We found no evidence that annualized serum metabolite slopes added held-out predictive value beyond landmark diagnosis, memory, and APOE ε4 in this sample. The PC aa C42:1 comparison was close to zero, including when evaluation was restricted to participants with MCI. This finding is conditional on the available sample and fitted models. Only 26 progression events were available for development, which limited our ability to estimate incremental effects or identify useful features. The results cannot distinguish between an absence of additional prognostic information and insufficient development data to identify it.

The distinction between biological association and incremental prediction is well established in biomarker research and prediction-model assessment (Collins et al., 2024; Van Calster et al., 2019; Wolff et al., 2019). Our contribution is a specific evaluation of annualized Quant 500 serum slopes under temporal separation and phase-based testing within ADNI. Metabolites can remain biologically informative even when a particular fitted model shows little incremental discrimination. In APP/PS1 mice, Cheng et al. (2022) reported coordinated alterations across fecal, serum, and cortical metabolomes and their relationships with the gut microbiota. In another neurological setting, Jiang et al. (2025) investigated cerebrospinal-fluid multi-omics, lipid metabolism, and functional outcomes after intracerebral hemorrhage. These studies provide biological context across systems and compartments; neither validates the prognostic value of serum slopes in human Alzheimer’s disease.

The earlier PC aa C42:1 screen and the present development sample overlap. Although the candidate was fixed before held-out performance evaluation, its selection was informed by prior outcome-related analyses and was not an independently prespecified hypothesis. This can favor an apparently promising analyte and carry selection-related optimism into subsequent evaluation. Separating model fitting from evaluation does not make the earlier discovery stage independent. We therefore regard PC aa C42:1 as a data-derived candidate, and its evaluation as hypothesis-generating until replicated outside the discovery data.

The development sample is the main constraint on interpretation. With 26 events, the clinical and candidate logistic models were vulnerable to unstable coefficient estimates, and selection among 88–598 slopes was particularly difficult. Sample-size requirements depend on model complexity, outcome frequency, and anticipated signal; penalization does not replace adequate development data (Riley et al., 2019). Under these conditions, cross-validation may favor a penalty that removes all metabolite features even when small incremental effects exist. Similarly, the random forest had too little event information to support a reliable assessment of high-dimensional non-linear relationships. Neither result should be read as evidence against multimetabolite or non-linear prognostic effects. The held-out bootstrap intervals condition on the fitted models and do not capture uncertainty from repeating development, candidate selection, or tuning in a new sample.

Outcome heterogeneity also limits interpretation. The combined endpoint counts cognitively normal-to-MCI or dementia transitions together with MCI-to-dementia transitions. These events arise from different starting stages and need not reflect the same biological process or prognosis. Syndromal diagnoses can also change in the presence of non-Alzheimer pathology, comorbidity, or measurement variability. A metabolite associated with one transition could have a weaker or different relationship with another, diluting an aggregate comparison. Because the endpoint was not biomarker-confirmed Alzheimer’s disease progression, the analysis cannot establish how serum slopes relate to Alzheimer-specific pathological change. The MCI-only analysis narrows the transition definition but does not make dementia etiology-specific.

Cohort composition differed substantially between development and evaluation. Nearly half of ADNI1 participants had MCI at the landmark, compared with more than four-fifths in ADNI-GO/2. Progression among cognitively normal participants was four of 117 in ADNI1 and 12 of 36 in ADNI-GO/2, whereas MCI-to-dementia progression was 22 of 108 and 38 of 233, respectively. The small cognitively normal evaluation subgroup and different transition rates make a pooled performance estimate sensitive to case mix. These observed differences should not be attributed to enrollment phase alone. The higher discrimination in the MCI-only evaluation shows that performance varies by the population being evaluated. That subgroup comparison still gave no evidence of additional discrimination from PC aa C42:1, but it cannot establish transportability to other clinics, stages, or populations.

Most participants contributed two metabolite observations. The annualized slope therefore usually represents a two-point rate of change rather than a detailed longitudinal trajectory. It can be affected by measurement error or transient changes at either visit and cannot distinguish sustained change from a temporary fluctuation. Richer non-linear patterns, timing of change, and within-person variability were not evaluated. Additional sampling could capture information missed by this representation, and the present findings should not be generalized to all longitudinal metabolomics approaches.

The clinical model showed imperfect calibration in ADNI-GO/2. A positive calibration intercept and a slope below one indicated that the transported probabilities did not reliably represent absolute risk in this cohort. Brier-score differences therefore combine discrimination with calibration error and should not be attributed solely to the information added by metabolites. Average precision is based on ranking and is unchanged by a strictly increasing recalibration that preserves that ranking. Its interpretation nevertheless depends on event prevalence and case mix, and it does not establish clinical benefit. Neither secondary metric establishes the clinical value of serial testing. No recalibration was performed in the held-out cohort (Saito and Rehmsmeier, 2015; Van Calster et al., 2019).

Strengths include temporal ordering of predictors and outcomes, a fixed outcome horizon, preprocessing and tuning confined to ADNI1, and paired evaluation of model differences. The phase-separated ADNI-GO/2 assessment remains an internal-temporal evaluation within ADNI. Independent cohorts are still needed. The analysis also applied the March 2026 ADNI Data Advisory and confirmed that no affected metabolomics records entered the analytic cohort.

Other limitations concern the clinical benchmark and missing data. The primary model was deliberately compact and did not include age, sex, education, imaging, or established plasma biomarkers. Age and longitudinal ADNI-MEM sensitivity analyses did not materially change the PC aa C42:1 comparison, but this does not establish performance against a comprehensive contemporary prognostic model. Single median imputation does not represent uncertainty about missing metabolite values or address informative missingness. Median feature missingness was 0% in both cohorts, and the stricter availability analysis gave a similar comparison, but these checks cannot eliminate selection bias from requiring an estimable candidate slope and later diagnostic follow-up. Results may also depend on assay platform and specimen-processing procedures.

Further work should use larger development cohorts with more progression events, independent candidate discovery and validation, and enough repeated measurements to assess patterns beyond a linear slope. Outcomes should be defined for the intended clinical population and, where appropriate, supported by Alzheimer-specific biomarkers. The systems-level framework described by Li et al. (2026) is useful for organizing biomarker relationships across organs and diseases, but translation still requires validation of a specified clinical use. Comparative studies should assess calibration and decision benefit alongside discrimination and the burden of repeat sampling.

## 5 Conclusion

We found no evidence of incremental held-out predictive value from annualized serum metabolite slopes in the available ADNI sample. The small number of development events limits what can be concluded about the underlying prognostic information. These results support further evaluation in adequately sized development and independent validation cohorts and do not establish that serial metabolomics lacks clinical value.

## Data Availability

Participant-level ADNI and ADMC data are available to approved investigators through the ADNI Image and Data Archive under the applicable Data Use Agreement. Analysis code, aggregate statistical tables, metadata, and publication figures are supplied with this submission. Participant-level records are not redistributed.

## Group members of Alzheimer’s Disease Neuroimaging Initiative and Alzheimer’s Disease Metabolomics Consortium

Data used in preparation of this article were obtained from the Alzheimer’s Disease Neuroimaging Initiative (ADNI) database (adni.loni.usc.edu). As such, the investigators within the ADNI contributed to the design and implementation of ADNI and/or provided data but did not participate in the analysis or writing of this report. A complete listing of ADNI investigators can be found at: https://adni.loni.usc.edu/wp-content/uploads/how_to_apply/ADNI_Acknowledgement_List.pdf.

Data used in preparation of this article were generated by the Alzheimer’s Disease Metabolomics Consortium (ADMC). The ADMC investigators provided data but did not participate in the analysis or writing of this report. A complete listing of ADMC investigators is available at: https://sites.duke.edu/adnimetab/team/.
