# Supplementary material for: No evidence that serum metabolite slopes improve held-out prediction of clinical progression in the Alzheimer’s disease neuroimaging initiative
Source: Front Aging Neurosci. 2026 Oct 1;18:1942662. doi: 10.3389/fnagi.2026.1942662 (PMC13670211; doi:10.3389/fnagi.2026.1942662)
Supplement: Supplementary file 1 [file Supplementary_file_2.DOCX]

# Supplementary Methods

## Landmark record selection

The nearest valid record was selected by absolute distance from the target month, with examination date used to break ties. The landmark window was months 8-16, and the outcome window was months 30-42. The outcome record was required to occur after the landmark record.

## Slope construction

Participant-visit duplicates were averaged. Raw annualized slopes and log2-transformed annualized slopes were estimated by ordinary least squares using at least two measurements spanning at least 6 months. Feature eligibility was determined in ADNI1 only.

## Lasso tuning

We retained landmark diagnosis, landmark ADNI-MEM, and APOE ε4 carrier status without penalization and applied an L1 penalty only to metabolite **slopes. The penalty was** selected by five-fold stratified cross-validation in ADNI1 over fractions 1, 0.5, 0.25, 0.125, 0.0625, 0.03125, and 0.015625 of the training-fold lambda-max. **Imputation** and scaling were fitted within each training fold. Ties in mean validation AUROC **favored** the stronger penalty. Both panels selected fraction 1.0 and retained no metabolite **slopes. With only 26 development events, this selection does not establish absence of metabolite information. A comparison with** the four-value grid (1, 0.5, 0.25, and 0.10) **used identical folds and produced** the same selected penalties and held-out predictions (Table S15). Data Sheets **S6–S7 report fold-level scores.**

## Random-forest tuning

The **exploratory** random forest contained 300 trees with balanced class weights and square-root feature sampling. Three-fold stratified cross-validation selected unrestricted depth and minimum leaf size 5 from the **candidate** grid. **Its performance is descriptive because 26 development events provide limited support for 598 candidate slopes. Feature importance and model underperformance are not evidence for or against reproducible nonlinear metabolite effects.**

## Quality-control sensitivity

For PC aa C42:1, a plate factor was calculated as the cohort-specific overall study-pool median divided by the plate-specific study-pool median. Concentrations below the plate-specific lower limit of detection were set to missing before reconstructing the annualized **slope.**

## Bootstrap inference

We generated 5,000 **paired,** class-stratified bootstrap resamples of the 269 held-out participants, sampling 50 events and 219 nonevents with replacement within class. NumPy default_rng used seed 20260719; **all** negative-class draws were generated first, followed by **all** positive-class draws. **The** same indices **were used across** models and metrics. **Average precision was recalculated in** each **resample; the** 2.5th and 97.5th percentiles **defined its** interval. The **fitted** models **remained fixed. These** intervals **describe evaluation-sample uncertainty** conditional on the models and class **counts; they do not include model-development or feature-selection uncertainty.** Calibration is **descriptive. Brier scores reflect probability calibration as well as discrimination. Average precision depends on ranking and class prevalence and does not measure calibration or** clinical **benefit.** Data Sheet S2 contains the unrounded **full-cohort** estimates.

**MCI-only held-out evaluation**

**The post hoc subgroup was defined by MCI at the month-12 landmark in ADNI-GO/2. All models remained fitted to the original 225 ADNI1 participants with 26 events. We did not refit, retune, or recalibrate within MCI. We evaluated 233 held-out MCI participants, including 38 MCI-to-dementia events, using the same metric functions and 5,000 paired class-stratified resamples, each containing 38 events and 195 nonevents. Tables S16–S17 report model performance and paired differences. Table S18 reports diagnostic transitions by cohort and landmark diagnosis. Code S4 reproduces this analysis. The subgroup evaluates the transported models within MCI; it is not a separately developed MCI prognostic model.**

# Supplementary Tables

**Table S1. Development-set coefficients for the clinical and candidate models.**

| Model | Term | OR (95% CI) | p value |
| --- | --- | --- | --- |
| Clinical | Intercept | 0.04 (0.01 to 0.12) | <0.001 |
| Clinical | Landmark MCI | 0.76 (0.17 to 3.33) | 0.711 |
| Clinical | APOE ε4 carrier | 3.05 (1.09 to 8.55) | 0.034 |
| Clinical | Landmark ADNI-MEM (z) | 0.19 (0.09 to 0.44) | <0.001 |
| Clinical + PC aa C42:1 | Intercept | 0.04 (0.01 to 0.12) | <0.001 |
| Clinical + PC aa C42:1 | Landmark MCI | 0.77 (0.18 to 3.38) | 0.729 |
| Clinical + PC aa C42:1 | APOE ε4 carrier | 2.92 (1.03 to 8.24) | 0.044 |
| Clinical + PC aa C42:1 | Landmark ADNI-MEM (z) | 0.19 (0.08 to 0.44) | <0.001 |
| Clinical + PC aa C42:1 | PC aa C42:1 annualized slope (z) | 0.78 (0.49 to 1.26) | 0.318 |

ADNI-MEM and metabolite **slope** coefficients are expressed per derivation-set standard deviation. These coefficients describe the ADNI1-fitted models; held-out performance is the primary inference.

**Table S2. Internal cross-validation used for penalized-model and random-forest tuning.**

| Model | Tuning | Selected penalty | CV AUROC | Retained metabolites |
| --- | --- | --- | --- | --- |
| Clinical + PC-family lasso | Five-fold ADNI1 AUROC | 1.0 × training-fold lambda-max; clinical terms unpenalized | 0.8612 | 0 |
| Clinical + full Quant 500 lasso | Five-fold ADNI1 AUROC | 1.0 × training-fold lambda-max; clinical terms unpenalized | 0.8612 | 0 |
| Clinical + full Quant 500 random forest | Three-fold ADNI1 AUROC | Unrestricted depth; minimum leaf size 5 | 0.7883 | NA |

**Table S3. Coefficients from the PC-family lasso at the selected penalty.**

| Feature | Coefficient | Feature type |
| --- | --- | --- |
| Landmark MCI | -0.2806 | Clinical |
| APOE ε4 carrier | 1.1155 | Clinical |
| Landmark ADNI-MEM (z) | -1.6443 | Clinical |

**Table S4. Coefficients from the full Quant 500 lasso at the selected penalty.**

| Feature | Coefficient | Feature type |
| --- | --- | --- |
| Landmark MCI | -0.2806 | Clinical |
| APOE ε4 carrier | 1.1155 | Clinical |
| Landmark ADNI-MEM (z) | -1.6443 | Clinical |

All three benchmark clinical predictors were forced into each **partially penalized** lasso without penalization. No metabolite **slope** was retained at the selected penalty in either model.

**Table S5. Quant 500 feature classes.**

| Metabolite class | Total features | Eligible slopes |
| --- | --- | --- |
| Triglycerides | 242 | 242 |
| Ceramides/glycosphingolipids | 70 | 70 |
| Diglycerides | 41 | 41 |
| Acylcarnitines | 40 | 40 |
| Acyl-alkyl phosphatidylcholines | 38 | 38 |
| Diacyl phosphatidylcholines | 36 | 36 |
| Cholesteryl esters | 22 | 22 |
| Other Quant 500 metabolites | 31 | 22 |
| Amino acids | 20 | 20 |
| Sphingomyelins | 14 | 14 |
| Lysophosphatidylcholines | 14 | 14 |
| Bile acids | 13 | 11 |
| Other polar metabolites | 12 | 10 |
| Fatty acids | 12 | 10 |
| Biogenic amines | 8 | 3 |
| Organic acids | 7 | 3 |
| Steroid/related hormones | 4 | 2 |

**Table S6. Top 20 random-forest feature importances.**

| Feature | Importance |
| --- | --- |
| Landmark ADNI-MEM | 0.0867 |
| C6__C4_1_DC | 0.0153 |
| Landmark MCI | 0.0148 |
| PC_ae_C40_3 | 0.0115 |
| APOE ε4 carrier | 0.0109 |
| PC_ae_C40_2 | 0.0100 |
| beta_Ala | 0.0098 |
| Taurine | 0.0084 |
| PC_ae_C44_3 | 0.0076 |
| C4_1 | 0.0075 |
| C12_1 | 0.0074 |
| C16 | 0.0067 |
| lysoPC_a_C26_1 | 0.0065 |
| Cer_d16_1_22_0 | 0.0064 |
| C14_2 | 0.0063 |
| PC_ae_C36_1 | 0.0056 |
| PC_ae_C42_5 | 0.0055 |
| C3_OH | 0.0053 |
| lysoPC_a_C28_0 | 0.0053 |
| X3_IAA | 0.0052 |

Importance values are impurity-based **estimates** from the **exploratory** ADNI1-fitted **forest. They can be unstable in this sample** and do not establish replicable feature relevance.

**Table S7. Log2-transformed slope sensitivity analyses using the partially penalized L1 model.**

| Model | AUROC | Brier | Average precision | Retained metabolites |
| --- | --- | --- | --- | --- |
| Clinical + log2 PC aa C42:1 slope | 0.758 | 0.127 | 0.517 | NA |
| Clinical + full Quant 500 log2-slope lasso | 0.7584 | 0.1238 | 0.5303 | 0 materially nonzero (\|β\| ≥ 0.001) |

**Table S8. Plate- and lower-limit-of-detection-adjusted PC aa C42:1 sensitivity analysis.**

| Measure | Value |
| --- | --- |
| Derivation participants | 225 |
| Evaluation participants | 269 |
| Evaluation events | 50 |
| AUROC (95% CI) | 0.759 (0.677 to 0.834) |
| ΔAUROC versus clinical (95% CI) | 0.0006 (-0.0128 to 0.0164) |
| Brier score | 0.126 |
| Average precision | 0.519 |

**Table S9. Analysis settings.**

| Setting | Specification |
| --- | --- |
| Predictor window | ADNI baseline through month 12 |
| Landmark assessment | Nearest valid diagnosis and ADNI-MEM from months 8-16 |
| Outcome assessment | Nearest subsequent diagnosis from months 30-42, targeting month 36 |
| Progression definition | CN to MCI/dementia or MCI to dementia; syndromal rather than biomarker-confirmed AD progression |
| Slope requirement | At least 2 values spanning at least 6 months |
| Derivation cohort | ADNI1 |
| Held-out evaluation cohort | ADNI-GO and ADNI2; phase-separated internal-temporal evaluation, not external validation |
| Feature eligibility | >=50% nonmissing annualized slopes in ADNI1 and nonzero variance |
| Missing slopes | Median imputation estimated from ADNI1 training data within each model fit |
| Scaling | Continuous predictors standardized from ADNI1 within each model fit |
| lasso | Clinical predictors forced in without penalty; L1 penalty applied only to metabolite **slopes** |
| Lasso tuning | Five-fold stratified ADNI1 cross-validation over fractions of training-fold lambda_max |
| Random forest tuning | Three-fold stratified ADNI1 cross-validation; 300 trees; balanced class weights |
| Class imbalance | No outcome resampling for logistic/lasso models; random forest used balanced class weights |
| Uncertainty | 5,000 paired class-stratified bootstrap samples in held-out ADNI-GO/2 |
| Random seed | 20260719 |
| Lasso penalty fractions | 1, 0.5, 0.25, 0.125, 0.0625, 0.03125, 0.015625 |
| Average-precision interval | 2.5th and 97.5th percentiles from the same 5,000 class-stratified held-out resamples; fitted models fixed |
| MCI-only held-out sensitivity | **Post hoc restriction at month-12 landmark; 233 participants, 38 events; no refitting, retuning, or recalibration; 5,000 paired stratified resamples.** |

**Table S10. Missingness summary for eligible slope features in the derivation and evaluation cohorts.**

| Cohort | Median missing | IQR | >10% missing | >25% missing | Maximum missing |
| --- | --- | --- | --- | --- | --- |
| ADNI1 derivation | 0% | 0-0% | 16 | 7 | 43.6% |
| ADNI-GO/2 evaluation | 0% | 0-0% | 15 | 9 | 98.9% |

**Table S11. Sensitivity to a stricter development-set feature-availability threshold.**

| Threshold | Eligible | Retained metabolites | AUROC | Brier | Avg. precision |
| --- | --- | --- | --- | --- | --- |
| 50% | 598 | 0 | 0.758 | 0.124 | 0.530 |
| 75% | 591 | 0 | 0.758 | 0.124 | 0.530 |

**Table S12. Feasibility of the alternative 0-6-month metabolite slope window.**

| Window | Dev n | Dev events | Eval n | Eval events | Fit | Reason |
| --- | --- | --- | --- | --- | --- | --- |
| 0-6 months | 5 | 1 | 21 | 11 | No | Insufficient development data |
| 0-12 months | 225 | 26 | 269 | 50 | Yes | Primary window |

**Table S13. Clinical-predictor sensitivity analyses incorporating age and longitudinal ADNI-MEM change.**

| Model | Dev n | Dev events | Eval n | Eval events | AUROC | Brier | Avg. precision |
| --- | --- | --- | --- | --- | --- | --- | --- |
| Clinical + age | 225 | 26 | 269 | 50 | 0.758 | 0.129 | 0.534 |
| Clinical + age + PC aa C42:1 | 225 | 26 | 269 | 50 | 0.757 | 0.133 | 0.511 |
| Longitudinal clinical | 210 | 26 | 179 | 30 | 0.746 | 0.118 | 0.493 |
| Longitudinal clinical + PC aa C42:1 | 210 | 26 | 179 | 30 | 0.745 | 0.119 | 0.497 |

**Table S14. Sensitivity analysis retaining diagnostic worsening occurring before the month-36 assessment window.**

| Model | Dev n | Dev events | Eval n | Eval events | AUROC | Brier | Avg. precision |
| --- | --- | --- | --- | --- | --- | --- | --- |
| Clinical | 225 | 26 | 269 | 52 | 0.754 | 0.13 | 0.527 |
| Clinical + PC aa C42:1 | 225 | 26 | 269 | 52 | 0.754 | 0.133 | 0.511 |

**Table S15. Comparison of the four-value and seven-value lasso penalty grids.**

| Panel | Grid | Selected fraction | CV AUROC | Retained metabolites | Held-out AUROC |
| --- | --- | --- | --- | --- | --- |
| PC-family | Four values | 1.0 | 0.8612 | 0 | 0.758 |
| PC-family | Seven values | 1.0 | 0.8612 | 0 | 0.758 |
| Full Quant 500 | Four values | 1.0 | 0.8612 | 0 | 0.758 |
| Full Quant 500 | Seven values | 1.0 | 0.8612 | 0 | 0.758 |

Both grids used the same five ADNI1 folds and seed 20260719. Each panel produced identical held-out predictions under the two grids (maximum absolute difference, 0). Held-out Brier score was 0.124 and average precision was 0.530 for every row. No held-out data were used to select penalties.

**Table S16. Held-out model performance in participants with MCI at the landmark.**

| Model | AUROC (95% CI) | Brier score (95% CI) | Average precision (95% CI) |
| --- | --- | --- | --- |
| Clinical | **0.860 (0.791 to 0.921)** | **0.096 (0.081 to 0.112)** | **0.608 (0.470 to 0.764)** |
| Clinical + PC aa C42:1 | **0.861 (0.796 to 0.917)** | **0.099 (0.084 to 0.115)** | **0.588 (0.454 to 0.744)** |
| PC-family lasso (exploratory) | **0.860 (0.791 to 0.921)** | **0.096 (0.081 to 0.112)** | **0.608 (0.470 to 0.764)** |
| Full-panel lasso (exploratory) | **0.860 (0.791 to 0.921)** | **0.096 (0.081 to 0.112)** | **0.608 (0.470 to 0.764)** |
| Full-panel random forest (exploratory) | **0.774 (0.705 to 0.837)** | **0.124 (0.118 to 0.130)** | **0.341 (0.264 to 0.464)** |

**All rows use the same 233 MCI participants and 38 MCI-to-dementia events. The original ADNI1 models were applied without refitting or recalibration. Intervals condition on the fitted models and subgroup class counts. Calibration intercept/slope: Clinical, 0.388/1.203; Clinical + PC aa C42:1, 0.421/1.157; Full-panel random forest (exploratory), -0.136/2.024. Both lasso models have the clinical-model calibration values. Full-precision values are provided in the CSV.**

**Table S17. Paired performance differences in the held-out MCI subgroup.**

| Model | ΔAUROC (95% CI) | ΔBrier (95% CI) | Δ average precision |
| --- | --- | --- | --- |
| Clinical + PC aa C42:1 | **0.0009 (-0.0159 to 0.0215)** | **0.0027 (-0.0004 to 0.0059)** | **-0.020** |
| PC-family lasso (exploratory) | **0.0000 (0.0000 to 0.0000)** | **0.0000 (-0.0000 to 0.0000)** | **0.000** |
| Full-panel lasso (exploratory) | **0.0000 (0.0000 to 0.0000)** | **0.0000 (-0.0000 to 0.0000)** | **0.000** |
| Full-panel random forest (exploratory) | **-0.0855 (-0.1608 to -0.0058)** | **0.0283 (0.0126 to 0.0430)** | **-0.267** |

**The clinical model is the reference. Positive ΔAUROC and Δ average precision and negative ΔBrier favor the candidate model. Both lasso models exactly reproduce the clinical predictions. Their zero intervals reflect identical fitted predictions and do not quantify uncertainty about potential effects under redevelopment. The random forest is exploratory.**

**Table S18. Diagnostic transitions by cohort and landmark diagnosis.**

| Cohort | Landmark diagnosis | N | Progression n (%) | Target CN / MCI / dementia, n |
| --- | --- | --- | --- | --- |
| ADNI1 | **Cognitively normal** | **117** | **4 (3.4%)** | **113 / 3 / 1** |
| ADNI1 | **MCI** | **108** | **22 (20.4%)** | **4 / 82 / 22** |
| ADNI-GO/2 | **Cognitively normal** | **36** | **12 (33.3%)** | **24 / 11 / 1** |
| ADNI-GO/2 | **MCI** | **233** | **38 (16.3%)** | **10 / 185 / 38** |

**CN, cognitively normal; MCI, mild cognitive impairment. Progression is CN-to-MCI or dementia for those initially CN and MCI-to-dementia for those with MCI at the landmark. Reversion to CN is classified as no progression. The outcome is syndromal and is not restricted to biomarker-confirmed Alzheimer’s disease.**

# Supplementary Figures

**Figure S1. Metabolite slopes retained by the exploratory full Quant 500 lasso. No metabolite slope was retained at the selected penalty; clinical predictors were unpenalized. This describes selection in the 26-event development sample and does not demonstrate absence of prognostic signal.**

**Figure S2. Composition of the eligible Quant 500 slope set. Bars show the number of annualized slopes meeting the ADNI1 availability and variance criteria within each broad metabolite class.**

**Figure S3. Held-out precision-recall curves in ADNI-GO/2. The clinical model and both exploratory lasso models share one curve because their predictions were identical. Separate curves show the PC aa C42:1 model and exploratory random forest. The dashed line denotes event prevalence. Average precision is descriptive and does not establish clinical benefit.**

# Supplementary Data Sheets

- Data Sheet S1. Full feature-level missingness in the ADNI1 derivation and ADNI-GO/2 evaluation cohorts (CSV).
- Data Sheet S2. **Held-out** performance for **the clinical and candidate models and the exploratory lasso and random-forest** models (CSV).
- Data Sheet S3. PC-family lasso coefficient table (CSV).
- Data Sheet S4. full Quant 500 lasso coefficient table (CSV).
- Data Sheet S5. Top 30 random-forest feature importances (CSV).
- Data Sheet S6. Fold-level ADNI1 validation AUROC for every candidate in the seven-value primary lasso grid (CSV).
- Data Sheet S7. Fold-level ADNI1 validation AUROC for the comparison of both penalty grids (CSV).
- Supplementary Code S1. primary fixed-landmark analysis code (Python).
- Supplementary Code S2. **Sensitivity** analyses for time-window feasibility, missingness, age, longitudinal clinical change, and early-progressor outcome ascertainment (Python).
- Supplementary Code S3. Reproducibility analysis comparing the four-value and seven-value lasso grids (Python).

**Supplementary Code S4. Held-out MCI-only sensitivity analysis using the original ADNI1 fitting procedures and source data (Python).**
